# Vaccination Coverage Among Children in Kindergarten — United States, 2013–14 School Year

**Published:** 2014-10-17

**Authors:** Ranee Seither, Svetlana Masalovich, Cynthia L Knighton, Jenelle Mellerson, James A. Singleton, Stacie M. Greby

**Affiliations:** 1 Immunization Services Division, National Center for Immunization and Respiratory Diseases, CDC; 2 Carter Consulting, Inc.

State and local vaccination requirements for school entry are implemented to maintain high vaccination coverage and protect schoolchildren from vaccine-preventable diseases (1). Each year, to assess state and national vaccination coverage and exemption levels among kindergartners, CDC analyzes school vaccination data collected by federally funded state, local, and territorial immunization programs. This report describes vaccination coverage in 49 states and the District of Columbia (DC) and vaccination exemption rates in 46 states and DC for children enrolled in kindergarten during the 2013–14 school year. Median vaccination coverage was 94.7% for 2 doses of measles, mumps, and rubella (MMR) vaccine; 95.0% for varying local requirements for diphtheria, tetanus toxoid, and acellular pertussis (DTaP) vaccine; and 93.3% for 2 doses of varicella vaccine among those states with a 2-dose requirement. The median total exemption rate was 1.8%. High exemption levels and suboptimal vaccination coverage leave children vulnerable to vaccine-preventable diseases. Although vaccination coverage among kindergartners for the majority of reporting states was at or near the 95% national *Healthy People 2020* targets for 4 doses of DTaP, 2 doses of MMR, and 2 doses of varicella vaccine (2), low vaccination coverage and high exemption levels can cluster within communities.* Immunization programs might have access to school vaccination coverage and exemption rates at a local level for counties, school districts, or schools that can identify areas where children are more vulnerable to vaccine-preventable diseases. Health promotion efforts in these local areas can be used to help parents understand the risks for vaccine-preventable diseases and the protection that vaccinations provide to their children.

Federally funded immunization programs assess vaccination coverage among children entering kindergarten each school year. Health departments, school nurses, or school personnel assess the vaccination and exemption status, as defined by state and local school requirements, of a census or sample of kindergartners enrolled in public and private schools. Among the 49 states and DC reporting vaccination coverage data, 42 used their immunization information system (IIS) as at least one source of data for their school assessment. The type of school survey varied among the 49 states and DC reporting either vaccination coverage or exemption: 38 reported using a census of kindergartners; nine a sample of schools, kindergartners, or both; one a voluntary response of schools; and two a mix of methods. Two states used a sample to collect vaccination coverage data and a census to collect exemption data. Four states changed their type of survey from the previous school year.† Data from the public and private school vaccination assessments were aggregated by state and DC immunization programs and sent to CDC.§ Vaccination coverage data were provided for 4,252,368 kindergartners included in reports from 49 states and DC, and exemption data were provided for 3,902,571 kindergartners included in reports from 46 states and DC.

All estimates of coverage and exemption rates were adjusted based on the type of survey conducted and response rates, using data aggregated at school or county level as appropriate and available, unless otherwise noted.¶ Vaccination requirements for school entry, as reported to CDC by the federally funded immunization programs, varied.** Kindergartners were considered up-to-date for any single vaccine if they had received all of the doses of that vaccine required for school entry in their jurisdiction. Nine states considered kindergartners up-to-date only if they had received all of the doses for all vaccines required for school entry in their jurisdiction.†† Of the 49 states and DC reporting vaccination coverage, 13 met CDC standards for school assessment methods in 2013–14.§§

Among the 49 states and DC that reported 2013–14 school vaccination coverage, median 2-dose MMR vaccination coverage was 94.7% (range = 81.7% in Colorado to ≥99.7% in Mississippi); 23 reported coverage ≥95% (Table 1), and eight reported coverage <90% (Table 1, Figure). Median local requirement for DTaP vaccination coverage was 95.0% (range = 80.9% in Colorado to ≥99.7% in Mississippi); 25 reported coverage ≥95%. Median 2-dose varicella vaccination coverage among the 36 states and DC requiring and reporting 2 doses was 93.3% (range = 81.7% in Colorado to ≥99.7% in Mississippi); nine reported coverage ≥95%.

Among the 46 states plus DC reporting 2013–14 school vaccination exemption data, the percentage of kindergartners with an exemption was <1% for eight states and ≥4% for 11 states (range = <0.1% in Mississippi to 7.1% in Oregon), with a median of 1.8% (Figure; Table 2). Two states reported increases over the previous school year of ≥1.0 percentage point: Kansas (1.5 percentage points) and Maine (1.2 percentage points). One state reported a decrease of ≥1.0 percentage points: West Virginia (1.0 percentage point). Where reported separately, the median rate of medical exemptions was 0.2% (range = <0.1% in eight states [Alabama, Arkansas, Colorado, Delaware, Georgia, Hawaii, Mississippi, and Nevada] to 1.2% [Alaska and Washington]). Where allowed and reported separately, the median rate of nonmedical exemptions was 1.7% (range = 0.4% in Virginia and DC to 7.0% in Oregon).

## Discussion

Most federally funded immunization programs continued to report high vaccination coverage and stable exemption rates among kindergartners during the 2013–14 school year compared with the 2012–13 school year, although 26 states and DC did not report meeting the *Healthy People 2020* target of 95% coverage for 2 doses of MMR vaccine. Although high levels of vaccination coverage by state are reassuring, vaccination exemptions have been shown to cluster geographically (3,4), so vaccine-preventable disease outbreaks can still occur where unvaccinated persons cluster in schools and communities (5).

School vaccination coverage assessment is used to assess state or local-level school vaccination requirements. Eighteen states provide local-level data online, helping to strengthen immunization programs, guide vaccination policies, and inform the public.¶¶ Local-level school vaccination and exemption data can be used by health departments and schools to focus vaccine-specific interventions and health communication efforts in a school or local area with documented low vaccination coverage or high exemption rates. Where expanded health communication strategies or other interventions are implemented, continued assessment and reporting can be used to facilitate program improvement.

To be most effective, accurate and reliable estimates of vaccination coverage and exemptions are needed. Use of appropriate sampling and survey methods can improve the usefulness of data for local use and comparability of estimates across school, local area, state, and national levels to accurately assess vaccination coverage and track progress toward *Healthy People 2020* targets.

School vaccination coverage reporting can be labor intensive, involving education systems at the start of the school year, when they are busiest. School vaccination assessment systems can be linked to an IIS, allowing schools to review the vaccination status of individual children. During the 2013–14 school year, 36 of the 50 states and DC reported that they allowed schools to obtain provider-reported vaccination data from their IIS, and 14 reported using an IIS algorithm to determine vaccination status for at least some of the students in their school vaccination assessment. An example of how an IIS can be used to simplify school vaccination assessment is Tennessee’s Immunization Certificate Validation Tool, which compares a child’s record in the state IIS against Tennessee vaccination requirements for pre-school or school attendance, allowing vaccination providers and school nurses to quickly assess a schoolchild’s vaccination status. It produces an official Tennessee Immunization Certificate or a detailed failure report. Tools linking school vaccination assessment systems to IIS data provide access to provider-reported information, reduce the documentation burden on parents and vaccination providers, and lessen the workload required by the assessment process on schools and health departments.

What is already known on this topic?

To protect school children from vaccine-preventable disease, annual school vaccination assessments indicate vaccination coverage and exemptions from state vaccination requirements. Although state vaccination coverage is high and exemptions are low, undervaccination and exemptions cluster at a local level, where vaccine-preventable diseases might be easily transmitted.

What is added by this report?

In 49 states and the District of Columbia (DC), median vaccination coverage for three vaccines was 94.7% for the measles, mumps, and rubella vaccine, 95.0% for varying local requirements for the diphtheria, tetanus toxoid, and acellular pertussis vaccine, and 93.3% for varicella vaccine among states with a 2-dose requirement. Of the 49 states and DC reporting vaccination coverage estimates, 27 did not report meeting the *Healthy People 2020* target of 95% coverage for 2 doses of measles, mumps, and rubella vaccine. Median exemption levels continue to be low overall (1.8%).

What are the implications for public health practice?

Local data are essential to controlling the spread of vaccine-preventable disease. Accurate and reliable school vaccination assessments can provide a unique opportunity for school and health departments to identify local areas of undervaccination, even at a school or classroom level, where the potential for disease transmission is higher. Health departments can use these data to identify schools and communities at higher risk for outbreaks and provide health communication interventions to protect school children and the community at large against vaccine-preventable diseases.

The findings in this report are subject to at least six limitations. First, not every state reported vaccination and exemption data. Second, vaccination and exemption status reflected the child’s status at the time of assessment. Reports might not be updated when parents submit amended school vaccination records after the required vaccines are received or an exemption is claimed. Third, a child with an exemption is not necessarily unvaccinated. More than 99% of the 2008–2009 birth cohorts who became kindergartners in 2013–14 received at least one vaccine in early childhood (6). An exemption might be provided for all vaccines even if a child missed a single vaccine dose or vaccine, or different exemptions might be provided for different vaccinations. A parent or guardian might choose to complete the required exemption paperwork if that is more convenient than having a child vaccinated or documenting a kindergartner’s vaccination history at school enrollment, which might be the reason for up to 25% of nonmedical exemptions (7–9).*** Fourth, methodology varied by reporting program or between school years for the same program. Methods and times for data collection differed, as did requirements for vaccinations and exemptions. Fifth, some programs (Delaware, Houston, Virginia, and Puerto Rico) were unable to provide detailed information needed to weight and analyze their data in the most statistically appropriate way, limiting the validity of their reported estimates. Finally, in adjusting data collected using school or student census methods to account for nonresponse, it was assumed that nonresponders and responders of the same school type had similar vaccination coverage and exemption rates.

State and local school vaccination assessments might detect local areas of undervaccination where disease transmission is more likely to occur. These data are most useful when the assessment is accurate and reliable. Use of statistically appropriate sampling methods and access to provider-reported vaccination data in an IIS can streamline the data collection process while providing accurate local-level data, allowing health departments to appropriately direct vaccination efforts during outbreaks of vaccine-preventable disease and identify schools and communities potentially at higher risk for vaccine-preventable disease transmission. Accurate local-level data can also be used by health departments and schools to focus health communication and other interventions that protect children and the community at large against vaccine-preventable diseases.

## Figures and Tables

**FIGURE:**
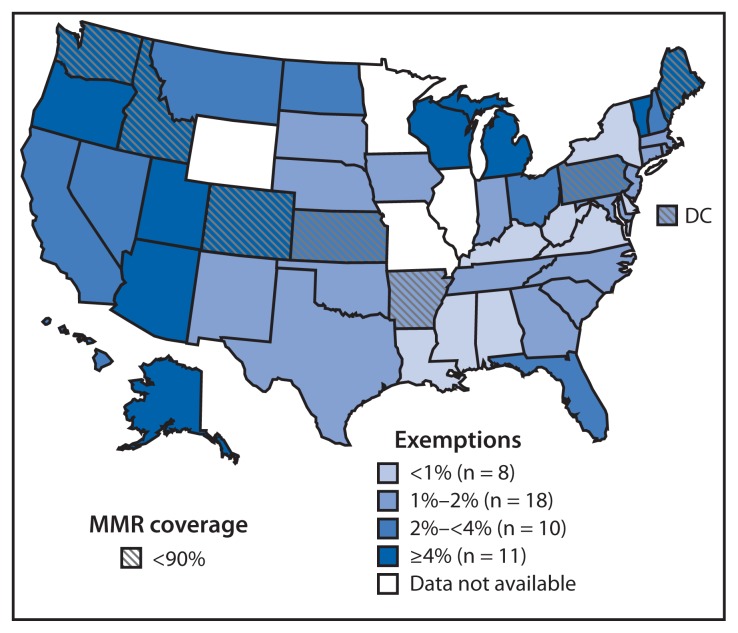
Estimated percentage of children enrolled in kindergarten who have been exempted from receiving one or more vaccines* and with <90% coverage with 2 doses of measles, mumps, and rubella (MMR) vaccine — United States, 2013–14 school year * Exemptions might not reflect a child’s vaccination status. Children with an exemption who did not receive any vaccines are indistinguishable from those who have an exemption but are up-to-date for one or more vaccines.

**TABLE 1:** Estimated vaccination coverage,* by state/area and vaccination among children enrolled in kindergarten — United States, 2013–14 school year

State/Area	Kindergarten population†	Total surveyed	Proportion surveyed (%)	Type of survey conducted§	MMR¶	DTaP**	Varicella	

							1 dose	2 doses
			
					(%)	(%)	(%)	(%)
Alabama††	76,927	76,927	100.0	Census	≥92.0	≥92.0	≥92.0	NReq
Alaska§§	10,222	946	9.3	Stratified 2-stage cluster sample	94.4	96.0		92.5
Arizona	89,606	85,861	95.8	Census	93.9	94.3	96.4	NReq
Arkansas	42,649	41,068	96.3	Census	86.5	83.3		85.4
California¶¶	548,606	533,680	97.3	Census	92.3	92.2	95.3	NReq
Colorado	69,904	350	0.5	Random sample	81.7	80.9		81.7
Connecticut††	40,978	40,978	100.0	Census	96.9	97.0		96.7
Delaware	11,997	1458	12.2	Stratified 2-stage cluster sample	≥96.4	≥96.4		≥96.4
District of Columbia††	7,856	7,856	100.0	Census	89.0	88.7		88.8
Florida††,***	233,797	233,797	100.0	Census	≥93.2	≥93.2		≥93.2
Georgia††	143,988	143,988	100.0	Census	≥94.0	≥94.0		≥94.0
Hawaii	20,056	1,074	5.4	Stratified 2-stage cluster sample	98.7	99.0	99.2	NReq
Idaho††	23,934	23,934	100.0	Census	88.2	88.0		86.5
Illinois††	163,316	163,316	100.0	Census	94.7	95.0	96.6	NReq
Indiana††	87,193	61,336	70.3	Census	92.9	81.8		90.2
Iowa	43,728	41,349	94.6	Census	≥91.0	≥91.0		≥91.0
Kansas§§,¶¶	41,107	11,931	29.0	Stratified 1-stage sample (Public), Census (Private)	86.9	87.6		85.5
Kentucky††	57,857	57,857	100.0	Census	92.6	93.9		91.9
Louisiana††	63,976	63,976	100.0	Census	96.8	98.3		96.1
Maine	15,441	12,716	82.4	Census	89.9	94.4	93.8	NReq
Maryland¶¶	75,659	73,349	96.9	Census	97.6	99.0	99.0	NReq
Massachusetts	79,894	78,188	97.9	Census	95.1	93.0		93.9
Michigan††	120,297	120,297	100.0	Census	97.5	94.8		93.0
Minnesota¶¶	72,087	70,972	98.5	Census	93.4	96.6		92.6
Mississippi††	45,719	45,719	100.0	Census	≥99.7	≥99.7		≥99.7
Missouri††	78,140	78,140	100.0	Census	95.5	96.0		94.6
Montana	12,855	12,259	95.4	Census	93.7	94.8		NReq
Nebraska¶¶	27,000	26,282	97.3	Census	96.6	96.8		94.9
Nevada	35,782	1,114	3.1	Stratified 2-stage cluster sample	95.6	94.4		93.6
New Hampshire††	13,240	13,240	100.0	Census	≥94.7	≥94.7		≥94.7
New Jersey	123,085	117,477	95.4	Census	≥96.8	≥96.8	≥96.8	NReq
New Mexico¶¶	30,725	830	2.7	Stratified 2-stage cluster sample	95.9	97.4		93.4
New York¶¶	240,318	240,318	100.0	Census	96.8	98.1	98.2	NReq
North Carolina	126,084	123,192	97.7	Census	98.8	98.7	99.7	NReq
North Dakota	9,780	9,397	96.1	Census (public) Stratified 2-stage cluster sample (private)	90.0	90.2		89.4
Ohio	150,000	138,820	92.5	Census	96.2	96.1		95.7
Oklahoma	57,377	40,929	71.3	Voluntary response	96.4	96.1		98.0
Oregon††	47,649	47,649	100.0	Census	93.2	93.3	94.3	NReq
Pennsylvania††,¶¶	151,253	151,253	100.0	Census	85.3	NReq†††		84.0
Rhode Island	11,521	11,421	99.1	Census	95.1	96.0		94.7
South Carolina	61,661	6,771	11.0	1-stage stratified sample	96.8	97.3	94.4	NReq
South Dakota††	12,566	12,566	100.0	Census	96.6	96.7		95.3
Tennessee	80,212	80,079	99.8	Census	≥94.9	≥94.9		≥94.9
Texas§§ (including Houston)	409,255	397,262	97.1	Census	97.5	97.2		97.2
Houston, Texas	36,254	1,856	5.1	2-stage cluster sample, nonrandom schools selection	91.9	90.4		90.4
Utah††	54,779	54,779	100.0	Census	98.5	98.1	99.6	NReq
Vermont††	6,771	6,771	100.0	Census	91.2	92.0		89.4
Virginia	105,692	4,287	4.1	2-stage cluster sample	93.1	98.3		91.3
Washington	89,165	78,924	88.5	Census	89.7	90.3		88.4
West Virginia	22,814	19,313	84.7	Census	96.1	96.5		95.5
Wisconsin¶¶	71,363	1,990	2.8	Stratified 2-stage cluster sample	92.6	96.3		91.2
Wyoming	NA	NA	NA	Not conducted				
*Median* §§§	*94.7*	*95.0*	*96.6*	*93.3*				
American Samoa	NA	NA	NA	Not conducted				
Guam	2,935	1,235	42.1	Stratified 2-stage cluster sample	88.4	92.8		NReq
Marshall Islands	NA	NA	NA	Not conducted				
Micronesia	NA	NA	NA	Not conducted				
N. Mariana Islands	725	725	100.0	Census	96.0	94.3		92.3
Palau	402	NA	NA	Not conducted				NReq
Puerto Rico	39,170	6,789	17.3	Stratified 2-stage cluster sample	94.3	91.3		91.4
U.S. Virgin Islands	1,612	731	45.3	Stratified 2-stage cluster sample	90.5	91.0		87.9

**Abbreviations:** MMR = measles, mumps, and rubella vaccine; DTaP = diphtheria and tetanus toxoids and acellular pertussis vaccine; NA = not available; NReq = not required for school entry.

* Estimates are adjusted for nonresponse and weighted for sampling where appropriate, except where complete data were unavailable. Percentages for Delaware, Houston, Virginia, and Puerto Rico are approximations. Estimates based on a completed vaccine series (i.e., not antigen-specific) are designated by use of the ≥ symbol.

† The kindergarten population is an approximation provided by each state/area.

§ Sample designs varied by state/area: census = all schools (public and private) and all children within schools were included in the assessment; simple random = a simple random sample design was used; mixed design = a census was conducted among public schools, and a random sample of children within the schools were selected; 1-stage or 2-stage cluster sample = schools were randomly selected, and all children in the selected schools were assessed (1-stage) or a random sample of children within the schools were selected (2-stage); voluntary response = a census among those schools that submitted assessment data.

¶ Most states require 2 doses; Alaska, California, New York, and Oregon require 2 doses of measles, 1 dose of mumps, and 1 dose of rubella vaccine.

** Pertussis vaccination coverage might include some DTP (diphtheria and tetanus toxoids and pertussis vaccine) vaccinations if administered in another country or if a vaccination provider continued to use DTP after 2000. Most states require 4 doses of DTaP vaccine; 5 doses are required for school entry in Colorado, District of Columbia, Hawaii, Idaho, Indiana, Iowa, Kansas, Massachusetts, Minnesota, New Jersey, New Mexico, North Carolina, North Dakota, Oregon, Rhode Island, Tennessee, Texas, Utah, Vermont, Washington, Northern Mariana Islands, Puerto Rico, and U.S. Virgin Islands; 3 doses are required by Nebraska and New York. Pertussis vaccine is not required in Pennsylvania.

†† The proportion surveyed is probably <100%, but is shown as 100% based on incomplete information about the actual current enrollment.

§§ Kindergarten coverage data were collected from a sample, and exemption data were collected from a census of kindergartners.

¶¶ Counts the vaccine doses received regardless of Advisory Committee on Immunization Practices recommended age and time interval; vaccination coverage rates shown might be higher than those for valid doses.

*** Does not include nondistrict-specific, virtual, and college laboratory schools, or private schools with fewer than 10 students.

††† Pertussis is not required in Pennsylvania; coverage for diphtheria and tetanus was 88.3%.

§§§ The median is the center of the estimates in the distribution. The median does not include Houston, Guam, the Commonwealth of the Northern Mariana Islands, Puerto Rico, and the U.S. Virgin Islands.

**TABLE 2:** Estimated number and percentage* of children enrolled in kindergarten with exemption(s) from vaccination, by state/area and type of exemption — United States, 2013–14 school year

State/Area	Medical exemptions†		Nonmedical exemptions†				Total exemptions†			
		
	No.	%	No. of religious exemptions	No. of philosophic exemptions	Total no.	%	Total no.	2013–14 (%)	2012–13 (%)	Percentage point difference
Alabama	70	<0.1	447	§	447	0.6	517	0.7	0.7	0.0
Alaska	119	1.2	421	§	421	4.1	539	5.3	5.6	−0.3
Arizona	175	0.2	¶	4,195	4,195	4.7	4,370	4.9	4.2	0.7
Arkansas	24	<0.1	135	333	468	1.1	493	1.2	1.1	0.1
California	1017	0.2	††	17,253	17,253	3.1	18,270	3.3	3.0	0.3
Colorado	0	<0.1	195	3,097	3,292	4.6	3,291	4.6	4.3	0.3
Connecticut	128	0.3	670	§	670	1.6	725	1.9	1.7	0.2
Delaware	9	<0.1	83	§	83	0.7	92	0.8	0.7	0.1
District of Columbia	85	1.1	33	§	33	0.4	118	1.5	1.6	−0.1
Florida	772	0.3	3,991	§	3,991	1.7	4,763	2.0	1.8	0.2
Georgia	143	<0.1	2,420	§	2,420	1.7	2,563	1.8	2.3	−0.5
Hawaii	0	<0.1	634	§	634	3.2	634	3.2	2.5	0.7
Idaho	89	0.4	147	1,304	1,451	6.1	1,540	6.4	5.9	0.5
Illinois**	NA				NA		NA	NA	6.1	NA
Indiana	348	0.4	727	§	727	0.8	1,075	1.2	1.3	−0.1
Iowa	205	0.5	521	§	521	1.2	726	1.7	1.7	0.0
Kansas	213	0.8	527	§	527	1.9	739	2.6	1.1	1.5
Kentucky	148	0.3	357	§	357	0.6	505	0.9	0.7	0.2
Louisiana	83	0.1	28	394	422	0.7	505	0.8	0.7	0.1
Maine	56	0.4	30	766	796	5.2	852	5.5	4.3	1.2
Maryland	244	0.3	513	§	513	0.7	758	1.0	1.0	0.0
Massachusetts	332	0.4	860	§	860	1.1	1,192	1.5	1.5	0.0
Michigan	573	0.5	1,250	5,226	6,476	5.4	7,049	5.9	5.9	0.0
Minnesota**	NA				NA		NA	NA	1.6	NA
Mississippi	17	<0.1	¶	§	NA		17	<0.1	<0.1	0.0
Missouri**	NA				NA		NA	NA	1.8	NA
Montana	36	0.3	426	§	426	3.3	463	3.6	3.5	0.1
Nebraska	158	0.6	307	§	307	1.1	465	1.7	1.7	0.0
Nevada	7	<0.1	724	§	724	2.0	731	2.0	2.5	−0.5
New Hampshire	49	0.4	328	§	328	2.5	377	2.8	2.5	0.3
New Jersey	262	0.2	1,741	§	1,741	1.4	2,003	1.6	1.4	0.2
New Mexico	72	0.2	277	§	277	0.9	349	1.1	0.4	0.7
New York	302	0.1	1,547	§	1,547	0.6	1,849	0.8	0.7	0.1
North Carolina	161	0.1	1,105	§	1,105	0.9	1,266	1.0	0.8	0.2
North Dakota	32	0.3	45	185	230	2.3	262	2.7	1.8	0.9
Ohio	369	0.2	††	††	2,681	1.8	3,050	2.0	2.0	0.0
Oklahoma	73	0.1	221	586	808	1.4	880	1.5	1.3	0.2
Oregon	62	0.1	3,331	††	3,331	7.0	3,393	7.1	6.5	0.6
Pennsylvania	510	0.3	1,133	1,419	2,552	1.7	3,062	2.0	2.0	0.0
Rhode Island	33	0.3	81	§	81	0.7	114	1.0	1.1	−0.1
South Carolina§§	83	0.1	772	§	772	1.2	855	1.4	NA	NA
South Dakota§§	21	0.2	199	§	199	1.6	220	1.8	1.8	0.0
Tennessee	132	0.2	773	§	773	1.0	906	1.1	1.2	−0.1
Texas (including Houston)	2,266	0.6	††	††	5,536	1.4	7,803	1.9	1.7	0.2
Houston	979	0.3	NA	NA	NA		979	0.3	0.9	−0.6
Utah	94	0.2	16	2,296	2,312	4.2	2,406	4.4	3.8	0.6
Vermont	11	0.2	13	399	412	6.1	423	6.2	6.1	0.1
Virginia	173	0.2	446	§	446	0.4	619	0.6	0.5	−0.5
Washington§§	1,035	1.2	311	2,866	3,177	3.6	4,212	4.7	4.6	0.1
West Virginia	35	0.2	¶	§			35	0.2	1.2	−1.0
Wisconsin	103	0.1	373	3,042	3,415	4.8	3,519	4.9	4.5	0.4
Wyoming	NA				NA		NA	NA	2.3	NA
*Median* ¶¶		*0.2*				*1.7*		*1.8*	*1.8*	*0.0*
American Samoa	NA				NA		NA	NA	NA	NA
Guam	0	<0.1	1	§	1	<0.1	1	<0.1	<0.1	0.0
Marshall Islands	NA				NA		NA	NA	NA	NA
Micronesia	NA				NA		NA	NA	NA	NA
N. Mariana Islands	0	0.0	0	0	0	0.0	0	0.0	0.1	−0.1
Palau	NA				NA		NA	NA	0.6	NA
Puerto Rico	0	<0.1	0	§	0	<0.1	0	<0.1	<0.1	0.0
U.S. Virgin Islands	0	0.0	17	§	17	1.1	17	1.1	0.6	0.5

**Abbreviation:** NA = not available (i.e., not collected or reported to CDC).

* Estimates are adjusted for nonresponse and sampling design where appropriate, except where complete data were unavailable. Percentages for Delaware, Houston, Virginia, and Puerto Rico are approximations.

† Medical and nonmedical exemptions might not be mutually exclusive. Some children might have both medical and nonmedical exemptions. Total exemptions is the number of children with an exemption. Temporary exemptions are included in the total for South Carolina, South Dakota, and Washington.

§ Exemptions because of philosophic reasons are not allowed.

¶ Exemptions because of religious reasons are not allowed.

** Lower bounds of the percentage of children with any exemptions, estimated using the individual vaccines with the highest number of exemptions are, for Illinois, 0.3% with medical exemptions, 1.0% with religious exemptions, and 1.3% for total exemptions, and for Missouri, 0.2% with medical exemptions, 1.6% with religious exemptions, and 1.8% for total exemptions. For Minnesota, the lower bounds of the percentage of children with any exemptions, estimated using the number of children exempt for all vaccines, are <0.1% with medical exemptions, 1.7% with religious exemptions, and 1.7% for total exemptions.

†† Religious and philosophic exemptions are not reported separately.

§§ Includes both temporary and permanent medical exemptions.

¶¶ The median is the center of the estimates in the distribution. The median does not include Houston, Guam, the Commonwealth of the Northern Mariana Islands, Puerto Rico, and the U.S. Virgin Islands.

## Footnotes

* *Healthy People 2020* objective IID-10.1 is based on 4 doses of DTaP vaccine. This report describes compliance with state regulations of 3, 4, or 5 doses of DTaP vaccine. Of the 49 states and DC, only Nebraska, New York, and Pennsylvania report <4 doses of DTaP vaccine. IID-10.2 sets a target of 95% of kindergartners receiving ≥2 doses of MMR vaccine. IID-10.5 sets a target of 95% of kindergartners receiving ≥2 doses of varicella vaccine.

† Alaska, Georgia, Missouri, and North Dakota.

§ Data from one local area (Houston) were reported separately and included in the data for the state of Texas. Oregon estimates included vaccination coverage and exemption data for children enrolled in public online homeschools. Pennsylvania included homeschool students in their public school data.

¶ Most of the programs that used complex sample surveys provided CDC with data aggregated at the school or county level for weighted analysis. Coverage and exemption data based on a reported census were adjusted for nonresponse using the inverse of the response rate, stratified by school type. For data collected using a complex sample design and with sufficient data provided, weights were calculated to account for sample design and adjusted for nonresponse. Where sufficient data were not available to account for the use of a stratified two-stage cluster sample design, data were analyzed as a stratified simple random sample (Delaware, Houston, Virginia, and Puerto Rico).

** Among the 49 reporting states and DC, all programs required 2 doses of a measles-containing vaccine, of which MMR is the only one available in the United States. For local requirements for DTaP vaccine, two required 3 doses, 27 required 4 doses, 20 required 5 doses, and one state did not require pertussis. For varicella vaccine, 13 required 1 dose, 36 required 2 doses, and 1 did not require varicella vaccination.

†† States reporting estimates based on receiving all doses of all vaccines required for school entry might have actual antigen-specific coverage estimates at least as high as the coverage for all required vaccines.

§§ CDC standards include use of a census or random sample of public and private schools or students, assessment using number of doses recommended by the Advisory Committee on Immunization Practices, assessment of vaccination status before December 31, collection of data by health department personnel or school nurses, validation if data are collected by school administrative staff, and documentation of vaccination from a health-care provider.

¶¶ Information available, by state, at the following websites: Alabama, http://www.adph.org/immunization/index.asp?id=761 ; Arizona, http://www.azdhs.gov/phs/immunization/statistics-reports.htm ; California, http://www.cdph.ca.gov/programs/immunize/pages/immunizationlevels.aspx ; Florida, http://www.floridahealth.gov/reports-and-data/immunization-coverage-surveys-reports/state-surveys.html ; Illinois, http://www.isbe.state.il.us/research/htmls/immunization.htm#immu ; Iowa, http://www.idph.state.ia.us/immtb/immunization.aspx?prog=imm&pg=audits ; Kansas, http://www.kdheks.gov/immunize/kindergarten_coverage.htm ; Kentucky, http://chfs.ky.gov/dph/epi/annual+immunization+school+and+childcare+survey.htm ; Michigan, http://www.michigan.gov/mdch/0,4612,7-132-2942_4911_4914_68361-321114--,00.html; Minnesota, http://www.health.state.mn.us/divs/idepc/immunize/stats/school/index.html ; New Jersey, http://www.state.nj.us/health/cd/stats.shtml ; North Dakota, www.ndhealth.gov/immunize/rates; Oregon, http://public.health.oregon.gov/preventionwellness/vaccinesimmunization/gettingimmunized/pages/schresources.aspx ; Texas, https://www.dshs.state.tx.us/immunize/coverage/default.shtm ; Utah, http://www.immunize-utah.org/statistics/utah%20statistics/immunization%20coverage%20levels/index.html ; Vermont, http://www.healthvermont.gov/hc/imm/immsurv.aspx ; Virginia, http://www.vdh.state.va.us/epidemiology/immunization/datamanagement/sisreports.htm ; Washington, http://www.doh.wa.gov/dataandstatisticalreports/schoolimmunization/datareports.aspx.

*** Tools are available to help parents manage vaccination records for their family; additional information available at http://www.cdc.gov/vaccines/parents/record-reqs/immuniz-records-child.html.
